# Apoptosis in Cellular Society: Communication between Apoptotic Cells and Their Neighbors

**DOI:** 10.3390/ijms17122144

**Published:** 2016-12-20

**Authors:** Yuhei Kawamoto, Yu-ichiro Nakajima, Erina Kuranaga

**Affiliations:** 1 Laboratory for Histogenetic Dynamics, Graduate School of Biological Sciences, Nara Institute of Science and Technology, Nara 630-0192, Japan; ykawamoto@cdb.riken.jp; 2 Laboratory for Histogenetic Dynamics, RIKEN Center for Developmental Biology, Kobe 650-0047, Japan; 3 Laboratory for Histogenetic Dynamics, Graduate School of Life Sciences, Tohoku University, Sendai 980-8578, Japan; yuichiro.nakajima.d2@tohoku.ac.jp; 4 Frontier Research Institute for Interdisciplinary Sciences, Tohoku University, Sendai 980-8578, Japan

**Keywords:** apoptosis, non-cell autonomous effects, engulfment, proliferation, mechanical force

## Abstract

Apoptosis is one of the cell-intrinsic suicide programs and is an essential cellular behavior for animal development and homeostasis. Traditionally, apoptosis has been regarded as a cell-autonomous phenomenon. However, recent in vivo genetic studies have revealed that apoptotic cells actively influence the behaviors of surrounding cells, including engulfment, proliferation, and production of mechanical forces. Such interactions can be bidirectional, and apoptosis is non-autonomously induced in a cellular community. Of note, it is becoming evident that active communication between apoptotic cells and living cells contributes to physiological processes during tissue remodeling, regeneration, and morphogenesis. In this review, we focus on the mutual interactions between apoptotic cells and their neighbors in cellular society and discuss issues relevant to future studies of apoptosis.

## 1. Introduction

In multicellular organisms, diverse cellular behaviors underlie the basis for constructing organs and maintaining homeostasis. These behaviors might appear to randomly occur; however, cells do communicate with each other in cellular society, and cellular behaviors are integrated to maintain tissue homeostasis and perform physiological functions. Apoptosis is a morphologically distinct form of cell death accompanied by a reduction in the cellular volume, condensation of the chromatin, and nuclear fragmentation. This particular cell death is one of the most fundamental cellular behaviors, removing unnecessary or potentially harmful cells by activating a genetically-encoded suicide program. Dysregulation of apoptosis mechanism causes disorders including developmental abnormalities, tumorigenesis, and autoimmune diseases.

Apoptosis has long been considered an autonomous phenomenon that does not actively affect surrounding cells. The clearance of apoptotic cells by phagocytic engulfment was discovered early in cell-death research, and the interaction between dying cells and engulfing cells was likely the sole communication assumed. With the progress of studies on apoptosis, however, the non-autonomous effects of apoptosis toward neighboring cells have been gradually revealed. Such apoptotic functions include the promotion of cell proliferation in the surrounding cells and morphogenetic changes of neighbors by generating mechanical forces. In addition to the effect of apoptosis on living cells, apoptosis itself can be non-autonomously controlled by neighboring cells; the process can be induced by engulfing cells, proliferating cells, and mechanical forces derived from cell crowding. Recent research clearly suggests that apoptotic cells actively communicate with neighboring cells, and their interactions play an important role in the maintenance of tissue homeostasis [1,2]. Studies using tractable genetic models, especially the fruit fly *Drosophila melanogaster*, have contributed to our understanding of this exciting field of research.

In this review, we focus on the cellular interactions between apoptotic cells and their neighbors in vivo from the perspective that apoptotic cells influence the surviving cells and vice versa, and discuss future directions in apoptosis research in cellular communities.

## 2. Apoptosis Signaling Pathway

**Figure 1:**
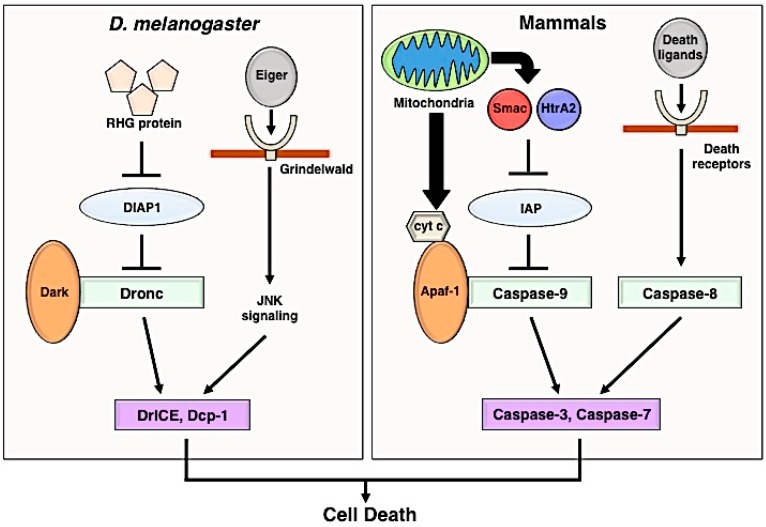
Schematic illustration of the apoptosis signaling pathway in *Drosophila* and mammals. The same colors and shapes represent functional homologous in both *Drosophila melanogaster* and mammals. In *Drosophila*, RHG proteins (Rpr, Hid, and Grim) produced by apoptotic stimuli inhibit the function of DIAP1 (*Drosophila* inhibitor of apoptosis protein 1). Dark (*apaf-1* homologue) forms a complex (apoptosome) with the initiator caspase Dronc. Effector caspases DrICE and Dcp-1 are activated by the apoptosome, and the activated effector caspases promote cell death. Eiger (*Drosophila* TNFα ortholog) induces the JNK (c-Jun N-terminal kinase)-mediated cell-death pathway through Grindelwald (TNF receptor). In mammals, Smac and HtrA2 released from mitochondria block the function of IAP (Inhibitor of apoptosis protein). Mitochondria also secretes cytochrome c (cyt c), and the apoptosome which is consisted of cyt c, Apaf-1, and pro-caspase-9 activates effector caspases, such as Caspase-3 and Caspase-7. Cell death via initiator caspase-8 requires the activation of death ligands and receptor signaling (TNFα-TNF receptor and Fas-Fas ligand). TNFα, tumor necrosis factor α.

Apoptosis is mediated by caspases, which are a family of cysteine proteases; its core mechanism is broadly conserved in multicellular organisms, from invertebrates to vertebrates (Figure 1). Caspases normally exist as inactive zymogens, and after being processed and separated into large and small subunits, inactive caspases acquire potential as proteases [3]. Caspases are subdivided into initiator caspases and effector caspases, both in mammals and *Drosophila*. Initiator caspases cleave specific substrates, including zymogens of effector caspases, which are subsequently activated by the cleavage. Active effector caspases break down intracellular proteins and induce apoptosis. Because such life-and-death decisions can be deleterious to cells, caspase signaling is carefully controlled at different points in the mechanism to avoid accidental upregulation. In mammals, the activation of initiator caspase-9 is regulated by inhibitor of apoptosis proteins (IAPs), which have E3 ubiquitin ligase activity, and if cells receive an apoptotic stimulus, IAP antagonists (SMAC and HtrA2) released from mitochondria bind to IAPs and promote their degradation [4,5,6,7]. Due to the degradation of IAPs, adaptor protein apoptosis-activating factor 1 (Apaf-1) forms a complex with pro-caspase-9, called an apoptosome, and promotes the activation of effector caspases [8].

In *Drosophila*, Reaper, Hid, and Grim (RHG) proteins are expressed upon apoptotic stimuli [9] and inactivate *Drosophila* inhibitor of apoptosis protein 1 (DIAP1) by degradation [10]. Once DIAP1 is degraded, the initiator caspase Dronc becomes active, resulting in the execution of apoptosis by activating the effector caspases DrICE and DCP-1 [11,12]. In addition to the intrinsic control, apoptosis is regulated by extrinsic signalings, such as the Fas-Fas ligand pathway and TNF-TNF receptor pathway (the TNFα ortholog Eiger and its receptor Grindelwald in *Drosophila* [13,14]). These signaling pathways also stimulate stress signaling cascades, such as the JNK pathway, which can induce cell death in a caspase-dependent and caspase-independent manner [15,16]. As shown in the next section, apoptotic or dying cells are rapidly engulfed by phagocytes and removed from tissues.

## 3. Engulfment and Apoptosis

In the animal body, unwanted or untoward cells undergo apoptosis and are rapidly engulfed by professional phagocytes, such as macrophages, or non-professional neighbors. During the engulfment process, apoptotic cells actively release secretory signals to recruit engulfing cells and express membrane proteins that engulfing cells can recognize. The former signals are called find-me signals, and the latter are eat-me signals [17]. Engulfing cells communicate with apoptotic cells through these signals and remove only dying cells in a process called apoptotic clearance. The engulfing cells or phagocytic pathways also contribute to the promotion of apoptosis or non-apoptotic cell removal by engulfment. The mutual interactions between apoptotic cells and engulfing cells are directed for the effective elimination of unnecessary cells.

### 3.1. Apoptosis Induces Engulfment

The efficient clearance of apoptotic cells is performed progressively through interactions with phagocytic cells via find-me and eat-me signals. Upon release from apoptotic cells, find-me signals are detected by phagocytes. Several find-me signals have been identified in the mammalian system, such as phospholipid lysophosphatidylcholine (LPC), sphingosine-1-phosphate (S1P) and CX3CL1/fractalkine, all of which are shown to work in a caspase-dependent manner [18,19]. Nucleotides like ATP and UTP released from apoptotic cells also function as find-me signals [20]. Once phagocytes are attracted toward apoptotic cells, they engulf the apoptotic cells. During this process, the phagocytes engulf apoptotic cells with phosphatidylserine (PS) exposed on their surface. The exposed PS is then recognized as an eat-me signal by engulfing macrophages, using secretory proteins MFG-E8 and Gas6 and receptor proteins Tim4, integrin and MER [17,21,22]. Thus, find-me and eat-me signals from apoptotic cells are necessary for effective and accurate engulfment.

**Figure 2:**
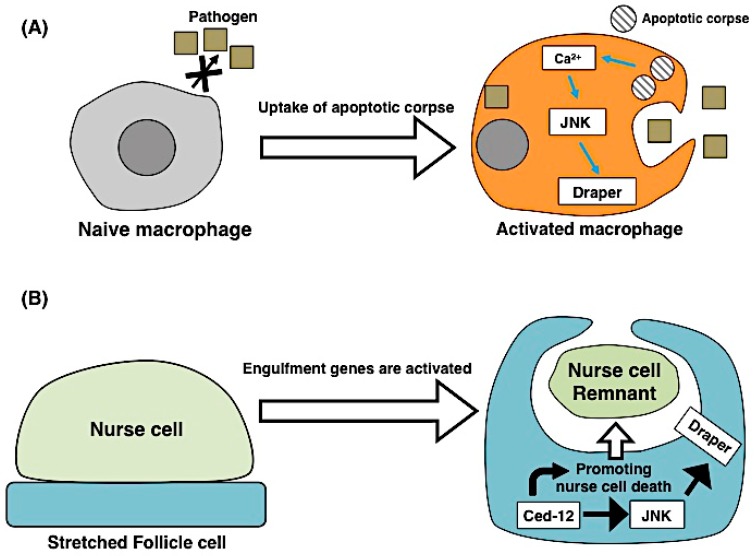
The mutual interaction between apoptotic cells and engulfing cells: (**A**) The process of macrophage priming. Naive macrophages cannot engulf pathogens. Apoptotic cells induce the non-autonomous activation of macrophages, and uptake of these cells leads to their transformation into mature macrophages; (**B**) Model for NC death in *Drosophila* oogenesis. Stretch follicle cells (FCs) contact nurse cell (NC) remnants during late oogenesis and non-autonomously promote NC death. In stretched FCs, the engulfment gene Draper becomes activated by the Ced-12-JNK signal. Ced-12 also contributes to NC death in parallel with the JNK-Draper pathway.

Apoptotic cells do not only attract macrophages but also contribute to the reprogramming of macrophage behaviors. A recent report by Weavers et al. suggests that apoptotic cells induce macrophage priming, which is a preliminary stimulation for activating the immune system (Figure 2A) [23]. During wound healing of the *Drosophila* embryonic epithelium, macrophage-like cells (hemocytes) normally detect wound sites and engulf apoptotic cells [24]. However, in the *H99* mutant that lacks developmental cell death, hemocytes do not engulf apoptotic cells. Interestingly, hemocytes in *H99* mutants fail to detect the wound site after tissue damage, suggesting that hemocytes are not capable of performing innate immune responses because of their lack of a memory of engulfment. This defect can be rescued if hemocytes incorporate apoptotic cells induced by ultraviolet radiation exposure. Molecularly, apoptotic corpses induce an increase in the calcium concentration and activation of JNK signaling in hemocytes, which affects the level of the receptor protein Draper that recognizes the exposed PS as a ligand [23]. This study elegantly shows that macrophage priming requires the uptake of apoptotic cells, as does immune priming, and is a novel example of the interaction between apoptotic cells and engulfing cells.

### 3.2. Engulfing Cells Contribute to Apoptosis

Engulfment by phagocytes was previously considered the last step of apoptosis. However, phagocytic cells or phagocytic pathways can actively promote cell death. Studies of cell death in *C. elegans* first revealed genetic evidence that engulfment genes contribute to apoptosis. Using hypomorphic mutants of ced-3, it was shown that additional mutations in engulfment genes significantly increase the number of surviving cells [25,26]. The expression of the engulfment receptor *ced-1* in engulfing cells rescues the defects in the killing function, suggesting that engulfment genes promote apoptosis in a non-cell-autonomous manner [25]. Chakraborty et al. further described the mechanism by which engulfment genes contribute to the actual killing procedure [27]. During asymmetric cell division of neurosecretory motor neuron neuroblasts (NSMnbs), the gradient of CED-3 is formed by mutual interaction with the engulfment receptor CED-1 on the surface of neighboring cells. The CED-3 distribution in NSMnbs becomes unequal during metaphase, and after cytokinesis, the CED-3 protein is unequally segregated and its concentration increases only in the daughter cell, which is destined to die [27]. This study suggests that engulfment pathways can promote apoptosis by providing the apoptotic potential in dying cells. Such cell death is also called “assisted suicide”, which is induced by coordinating canonical apoptotic genes and engulfment genes [28].

In addition to such assistance of apoptosis by phagocytic pathways, engulfing cells are directly involved in cell death. In the developmental ovary of *Drosophila*, germ line-derived nurse cells (NCs) undergo programmed cell death during oogenesis. The *Drosophila* ovary has few professional phagocytes; instead, neighboring epithelial follicle cells (FCs) engulf NC remnants [29]. The engulfment receptor Draper and JNK activity in the FCs are required for the clearance of NCs after starvation-induced cell death. Intriguingly, the overexpression of *draper* or the JNK pathway in FCs induce NC cell death, suggesting that FCs can trigger non-autonomous NC death [30]. A recent report by Timmons et al. showed that naturally occurring NC cell death is regulated by phagocytic machinery in FCs (Figure 2B) [31]. During late oogenesis, a population of FCs–the stretched FCs–surround NCs throughout the process of cell death until their removal by engulfment. When the engulfment genes *draper* and *Dced-12* or the JNK signaling pathway was blocked in the stretched FCs, NC death and removal were suppressed. Although NC cell death is not caspase-mediated apoptosis, the engulfment genes in the FCs non-autonomously control events associated with NC cell death, such as nuclear envelope permeabilization and DNA fragmentation. Furthermore, genetic ablation of the stretched FCs prevented the processes of NC cell death, suggesting that the FCs indeed non-autonomously control NC cell death and their removal. Such phagocytosis is known as “phagoptosis” and is regarded as primary phagocytosis that induces apoptosis in viable cells. Phagoptosis is also observed in human cultured microglia, which are brain macrophages that release nitric oxide (NO) and induce the exposure of PS in surrounding cells [32,33]. Altogether, these recent findings suggest that engulfing cells actively contribute to apoptotic or non-apoptotic cell death by utilizing the engulfment machinery.

## 4. Proliferation and Apoptosis

The number of cells in tissue is essential for controlling the size and shape of the tissue and is constantly monitored to maintain tissue homeostasis. To control cell numbers, cell proliferation and apoptosis are crucial cellular behaviors, and thus their balance must be tightly coordinated. For example, after tissue injury, when damaged cells undergo apoptosis, neighboring cells that are adjacent to the dying cells may proliferate and fill in the gaps left behind. Such additional proliferation is called compensatory proliferation or apoptosis-induced proliferation [34,35]. Furthermore, apoptosis can be non-autonomously induced by proliferating cells via sensing the difference in the metabolic status or cellular nature. In this section, we review the current understanding of the mechanism of apoptosis-induced proliferation and non-autonomous apoptosis triggered by proliferating cells.

### 4.1. Apoptosis Induces Proliferation

Compensatory proliferation or apoptosis-induced proliferation was first experimentally shown in *Drosophila* imaginal discs. Imaginal discs are larval primordial tissue of adult fly appendages, such as wings, eyes, and legs, which are composed of polarized epithelial cells. When massive apoptosis was induced by X-ray irradiation in the developing wing imaginal disc, the size and shape of the adult wings were nearly normal [36]. This study suggested that the tissue had a mechanism for recovering from a severe decrease in cell numbers. The molecular mechanism of compensatory proliferation has been investigated in “undead cells”, where apoptosis itself is blocked. Undead cells are generated in proliferating imaginal discs by either irradiation or the expression of pro-apoptotic genes when effector caspases are inhibited by the caspase inhibitor *p35.* Under such conditions, hyperplastic tissue overgrowth occurs because the undead cells continue to secrete mitogenic signals picked up by their neighboring cells. According to previous reports, undead cells in imaginal discs secrete mitogenic factors, Wnt homologue Wingless (Wg), BMP homologue Decapentaplegic (Dpp), and epidermal growth factor (EGF), which are necessary for triggering the proliferation of surrounding cells [37,38,39,40]. This additional proliferation requires the activation of an initiator caspase Dronc, and the subsequent activation of the stress signaling factors JNK and p53 [39,41,42]. Several factors discovered in this experimental system play an important role in regenerating processes. Two recent reports showed that reactive oxygen species (ROS) contribute to apoptosis-induced proliferation in both *Drosophila* and the zebrafish *Danio rerio*. In *Drosophila*, the importance of ROS has been revealed by the undead-cell system. Extracellular ROS are required for JNK activation via TNF pathway mediated by macrophage-like hemocytes [43]. Of note, ROS are necessary for regenerative responses induced in the eye imaginal disc. In adult zebrafish, ROS are detected in apoptotic cells after tail amputation, and ROS production contributes to JNK activation, which is required for apoptosis-induced proliferation during regeneration [44].

**Figure 3:**
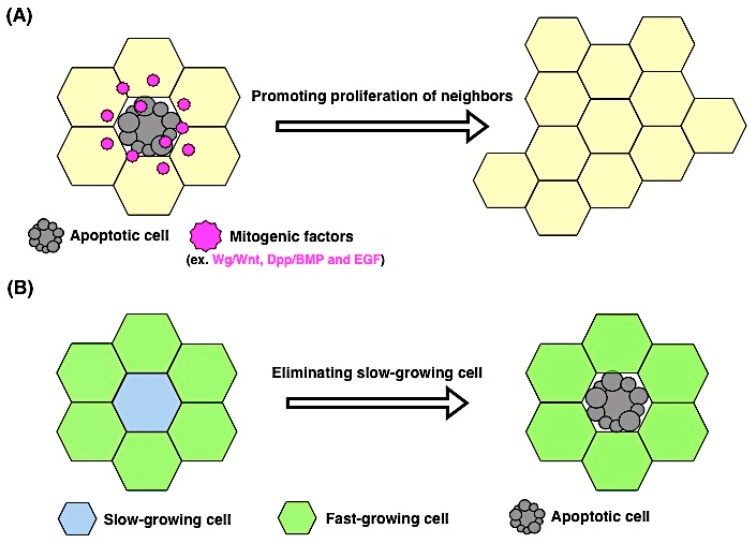
The control of cell numbers in tissue by non-autonomous effects of proliferation and apoptosis. (**A**) Apoptosis-induced proliferation. Apoptotic cells secrete mitogenic signals (e.g., Wnt, BMP and EGF) that induce the proliferation of neighboring cells (yellow hexagons) and contribute to tissue regeneration; (**B**) Proliferating cells induce non-autonomous apoptosis. Slow-growing cells are eliminated by their surrounding fast-growing cells. This event, called cell competition, is essential for the maintenance of tissue homeostasis.

Apoptosis-induced proliferation functions as a core for organ regeneration. In the intestines, the intestinal epithelium serves as a barrier and responds to environmental toxins and physical stress. Upon such stimulation, orchestrated regenerative responses initiate by coordinating the behaviors of intestinal stem cells and their neighbors. In the *Drosophila* gut, damaged epithelial cells (enterocytes) release cytokines Upd, Upd2, and Upd3 (*Drosophila interleukin-6* homolog). These cytokines activate JAK/STAT signaling in intestinal stem cells and its progenitors, resulting in the promotion of cell division of intestinal stem cells and the progenitors’ differentiation [45]. Such apoptosis-induced proliferation during regeneration is also observed in diverse taxa, including the head in *Hydra*, the tail in *Xenopus leavis*, and the liver in mice [1,46]. Secretory proteins from apoptotic cells drive cell proliferation in their neighbors, and although the proteins differ between organisms, Wnt, Notch, FGF, TGF-β, and production of prostaglandin E2 (PEG2) have been identified as candidates [47,48,49,50,51,52]. Overall, these reports support the notion that compensatory proliferation is initiated by secretory factors from apoptotic cells and reinforces the significance of the active participation of apoptosis in tissue regeneration (Figure 3A).

### 4.2. Proliferating Cells Contribute to Apoptosis

The close associations of proliferation and apoptosis can be bidirectional events, and indeed, apoptosis is non-autonomously induced by proliferating cells. One of the best-known examples is a competitive cellular interaction in which slow-growing cells are eliminated by fast-growing cells (Figure 3B). This phenomenon, called cell competition, was originally discovered in *Drosophila* during the study of dominant mutations of *Minute*, which encodes ribosomal proteins [53]. In heterozygous *Minute* flies, a developmental time delay occurs because of cell-autonomous reduction of the growth rate [53]. Interestingly, early-induced *Minute+/−* clones in a wild-type background are eliminated if surrounded by wild-type cells [54]. In this situation, the wild-type cells were the faster growing cells and the heterozygous *Minute*+/*−* cells the slower ones, suggesting that the elimination of *Minute+/−* cells occurs based on the difference in the cell proliferation rate. Later studies proved that the elimination of slowly growing cells is induced by non-autonomous apoptosis at the boundary of wild-type cells [55,56].

Similarly, *Drosophila* Myc homologue (*dMyc*) mutant cells are outcompeted by wild-type cells [57]. In a situation where the expression of *dMyc* is higher than in neighboring cells, even surrounding wild-type cells undergo apoptosis as a result of competition [58,59]. The decision for Myc-dependent cell competition is likely mediated via the membrane protein Flower, with different isoforms corresponding to different levels of *dMyc* [60]. In *dMyc*-overexpressing cells, glycolysis is also enhanced through the activation of *p53* to maintain genome stability and viability in cells. This evidence suggests that Myc-dependent cell competition requires the regulation of metabolic status mediated by p53 as highly proliferating cells [61]. Such competitive cellular interactions are also observed in mammals. In mosaic mouse embryos with cells with different levels of c-Myc, the cells expressing lower levels of c-Myc were eliminated via apoptosis [62]. Myc-dependent cell competition has also been confirmed in embryonic stem cells and heart development [63,64]. These studies suggest that cell competition is an evolutionarily conserved phenomenon in which non-autonomous apoptosis is induced by different proliferation rates or metabolic status.

Competitive-like cell interactions can determine life-and-death decisions between proliferating and non-proliferating cells. In the *Drosophila* pupal stage, replacement of the epithelium takes place in the abdomen, where larval epidermal cells (LECs) are removed by apoptosis and adult precursor cells (histoblasts) replace them [65]. Most apoptosis in LECs occurs at the proximity of histoblasts, and if the cell cycle in histoblasts is inhibited, proper caspase activation and subsequent apoptosis in neighboring LECs are blocked [66,67]. These results demonstrate that LEC apoptosis is non-autonomously induced by proliferating histoblasts via local cell-cell interactions. The molecular mechanism of non-autonomous LEC apoptosis remains unknown. During abdominal epithelial replacement, histoblasts extend specialized filopodia-like protrusions, called cytonemes, toward LECs [68]. Cytonemes are actin-rich structures involved in transporting signaling proteins [69]. Given that local cell–cell interactions trigger non-autonomous LEC apoptosis, secretory proteins mediated through cytonemes may be involved in this event. Such communications between proliferating cells and non-proliferating cells might be applied to interactions between stem cells and terminally differentiated cells in different organisms.

## 5. Force and Apoptosis

During development and homeostasis, cells in tissue interact physically with their neighbors via cell adhesions and the extracellular matrix. Such physical interactions are particularly essential for epithelial tissue where various morphological changes occur as a cell sheet while maintaining tissue integrity. Morphogenetic events need driving forces to change individual cell shapes and the shape of a tissue. Recent research progress suggests that apoptosis accompanying cell delamination or extrusion from the epithelium is one of the driving forces that directly influence neighboring cells by producing mechanical forces. Conversely, mechanical forces generated from cell-crowding non-autonomously induce live cell extrusion or apoptotic cell death. Thus, the relationship between force and apoptosis is closely interconnected in the cellular community, serving as a morphogenetic driver and a homeostatic mechanism.

### 5.1. Apoptosis Induces Mechanical Force

The first in vivo demonstration about force production from apoptotic cells came from the study of a tissue fusion event, dorsal closure in *Drosophila* embryogenesis. During dorsal closure, the lateral epidermis spreads and migrates dorsally, finally covering the dorsal surface. The amnioserosa, which is an extra embryonic tissue, initially occupying the most dorsal region, reduces its apical surface area, resulting in basal extrusion. Multiple forces from the retracting amnioserosa and the spreading lateral epidermis were suggested to contribute to the movement of the epidermal cells [70]. Toyama et al. found that apoptosis occurs in the constricting amnioserosa cells and further examined the possibility that apoptosis produces a mechanical force [71]. While the inhibition of apoptosis in aminoserosa cells by the expression of *p35* caused a delay in dorsal closure, the promotion of apoptosis by the pro-apoptotic gene *grim* accelerated the closure process. Laser cutting experiments demonstrated that epidermal cells are pulled by active mechanical forces derived from apoptotic amnioserosa cells [71]. Following this study, Muliyil et al. identified hierarchies in the apoptosis signaling cascade during dorsal closure and found that mitochondrial fragmentation is an initial event that is necessary for the extrusion of amnioserosa cells [72]. Taken together, these findings suggest that developmental apoptosis plays an active role in morphogenetic changes by generating mechanical forces.

Mechanical forces produced by apoptosis can contribute to epithelial sheet movement or collective cell migration during organogenesis. In the developing *Drosophila* male genitalia, the genital disc plate turns 360° and gives rise to part of the adult genital and anal structures [73,74]. It was shown that cell death mutants have abnormal rotation [75], but the relationship between apoptosis and the rotation movement remained unclear. Recent studies using a live imaging approach revealed local apoptosis during genitalia rotation and addressed its role in genitalia development. When apoptosis was inhibited by the caspase inhibitor *p35*, although the genital disc moved to some extent, it failed to attain complete 360° rotation, suggesting that apoptosis acts as a brake-release in rotation movement [76]. In addition, when apoptosis was genetically inhibited or promoted, the rotation speed decreased or increased respectively, depending on the manipulation [77]. Taken together, these findings suggest that local apoptosis regulates both the beginning of rotation and the rotation speed, acting as a key factor for the control of tissue movement.

**Figure 4:**
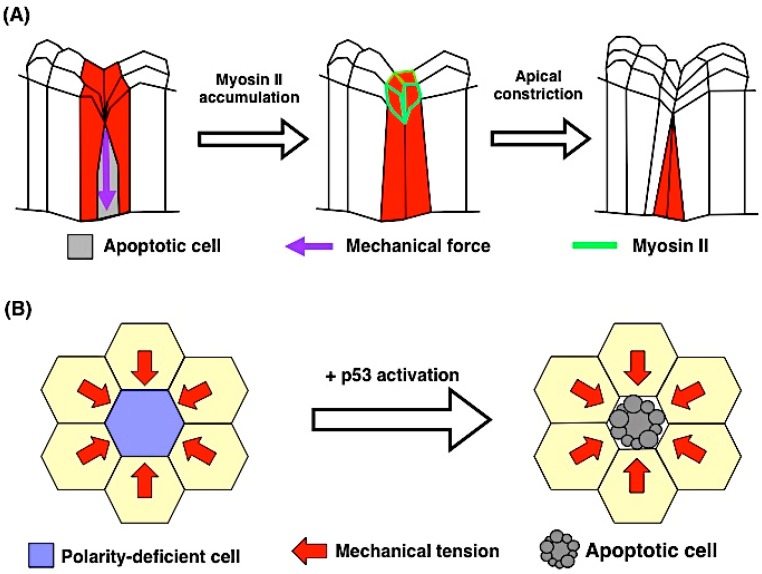
The interaction between apoptosis and mechanical force. (**A**) Apoptosis-induced morphogenetic changes in *Drosophila* leg joint formation. Apoptotic cells pull their neighbors toward the basal side during delamination at the presumptive joint area. In their neighboring cells, the actomyosin cable accumulates in the apical cortical area. The apical surface of surrounding cells undergoes apical constriction; (**B**) Model for mechanical tension-induced cell competition. In cultured mammalian epithelial cells, compaction by overcrowding induces apoptosis in polarity-deficient cells, which is mediated by the upregulation of *p53*. The yellow hexagons represent normal cells.

How does apoptosis produce active mechanical forces? A recent study addressed this issue using *Drosophila* leg joint formation as a model [78]. Each leg of adult *Drosophila* derives from a corresponding larval precursor, a leg imaginal disc. During leg disc development, the initial flat sheet structure creates several folds, and the tissue is finally separated by flexible joint structures [79]. It was previously shown that apoptosis occurs at the presumptive joint area, and when apoptosis was inhibited by *p35* in the leg disc, the formation of the joint was incomplete, resulting in an abnormal shape of the adult leg [80]. Monier et al. further investigated the process of joint formation and examined the hypothesis that apoptosis induces mechanical force that allows tissue reshaping during leg joint formation (Figure 4A) [78]. In apoptotic cells at the presumptive joint area, the apical surface deforms following the cell shrinkage. During this process, an actomyosin cable is formed in apoptotic cells, and the neighboring cells are pulled toward the apoptotic cells during delamination. An in silico model also confirmed that both apoptosis and the mechanical force derived from apoptotic cells are sufficient for attaining folding. These results suggest that the actomyosin cable contributes to the contraction of the apical surface of the apoptotic cells and the subsequent cell shape change of the neighboring cells, implying that the apoptotic signal non-autonomously triggers actomyosin contractility in neighboring cells and synergistic mechanical forces derived from delaminating cells and neighboring cells contribute to proper morphogenesis.

Such effects of apoptosis on mechanical force production are probably conserved throughout evolution. During cranial neural tube closure (NTC) in mice, large populations of cells undergo apoptosis [81]. In NTC formation, the neural plates move toward the midline and finally fuse to form the roof of the neural tube. According to Yamaguchi et al., in *apaf-1* or *caspase-3* mutants, the speed of the dorsal plate movement becomes delayed, and the fusion at the midline fails [82]. This evidence suggests an active role of apoptosis in dynamic tissue movement during NTC. Collectively, these studies imply that apoptotic cells directly influence the behavior of their neighboring cells by generating mechanical forces.

### 5.2. Mechanical Force Contributes to Apoptosis

In epithelial tissues, to avoid the breakdown of tissue integrity or hyperplasia, the number and quality of cells are controlled by multiple homeostatic mechanisms. Ordinarily, unwanted cells are eliminated by apoptosis and removed from the epithelial layer. However, recent studies suggest that both apoptotic and living cells are extruded from the epithelium by crowding-induced mechanical force. In the extrusion of cultured epithelial cells, apoptotic stimuli induce apical surface constriction by actomyosin-mediated contracting force [83]. It has been shown in tissue culture and zebrafish epithelia that the extrusion of apoptotic cells is controlled by the S1P signaling pathway upon apoptotic stimulation [84]. In contrast, in the developing fly notum epithelium and zebrafish epidermis, crowding-induced forces induce living cell extrusion or delamination, in addition to apoptotic extrusion. The dorsal region of the fly thorax, called the notum, consists of a single epithelial layer, and during metamorphosis, notum precursor cells divide without changing the overall tissue size, which can cause pressure in the tissue. In the developing notum, large populations of cells delaminate basally in the midline region and ultimately undergo cell death, probably because of anoikis. The rate of cell delamination increases under conditions where tissue growth is genetically promoted. Blocking of apoptosis did not seem to suppress the extrusion, suggesting that basal delamination is mechanistically distinct from apoptosis-mediated extrusion [85]. However, Levayer et al. contrastingly found that crowded conditions induced caspase activation in the midline of the notum, which precedes and is required for cell delamination. Their study supports the conclusion that cell delamination is induced by unknown apoptotic stimuli, probably through local tissue tension [86]. In zebrafish, both apoptotic and non-apoptotic live cells are extruded in an area of high cell density in the tail. To determine whether or not the rate of crowding-induced extrusion changes in proportion to cell density, Eisenhoffer et al. established an experimental cell-crowding system with cultured mammalian cells by adding mechanical pressure and found that, under the overcrowding condition, the extrusion of living cells was predominantly promoted [87]. Interestingly, live cell extrusion also requires S1P signaling, just like apoptotic extrusion, and defects of proper cell extrusion contribute to tumor progression [88]. Taken together, these findings suggest that increases in mechanical tension trigger cell extrusion in crowding tissue, which is regulated by both apoptotic and non-apoptotic stimuli.

What determines whether cell delamination is mediated in an apoptotic or non-apoptotic way? Wagstaff et al. proposed that compaction by overcrowding induces apoptotic cell death in cultured mammalian epithelial cells through p53 activation in the context of polarity-deficient cell competition (Figure 4B) [89]. Under these conditions, mechanical stress activates the actin cytoskeleton regulator ROCK, which leads to the elevation of p53 through active p38 [89,90]. However, how cell-crowding is sensed at the cell or tissue level and how mechanical forces induce caspase activation in vivo remain poorly understood. Clarifying the precise mechanism of cell extrusion in living animals is an important field that will provide mechanistic insights into diseases such as cancer.

## 6. Future Directions

In this review, we focused on apoptotic functions as a switch for initiating actions of neighboring cells during immune responses, regeneration, and morphogenetic changes. The progression of cell death is also non-autonomously induced by interplay with surrounding cells; as such, cell–cell interactions during apoptosis-induced events or non-autonomous apoptosis are essential for the maintenance of tissue homeostasis, and defects in these interactions can lead to diseases. Although we focused on direct interactions or close associations of apoptotic cells and their living neighbors, it is becoming clear that the effect of apoptosis is not restricted to the cell or within-tissue level. Indeed, local apoptosis in tissue can influence distant tissue and even the whole body. When the adult fly cuticle is damaged, ROS activation is promoted in enterocytes of the midgut, which is a remote organ from the wound site, causing enterocytes to undergo apoptosis. Apoptosis is thus involved in epithelial cell renewal by stimulating intestinal stem cell proliferation. Interestingly, if epithelial cell renewal after cuticle damage is blocked by the inhibition of enterocyte apoptosis, then flies exhibit lethality, probably due to defects in the damping lethal responses [91]. Such inter-organ communications via apoptosis has also been reported in tissue regeneration. Kashio et al. examined the systemic effects of local tissue damage in the wing disc epithelium and found that methionine metabolism changed in the fat body, which is a *Drosophila* adipose tissue. The altered methionine metabolism is required for wing disc tissue repair, suggesting that local apoptosis is linked to the fat body metabolism via systemic factors [92]. These findings suggest that apoptotic cells systematically influence homeostasis of the whole body and overall organism health.

Recent reports have implied potential crosstalk between individual cellular behaviors influenced by apoptotic cells. According to Hochreiter-Hufford et al., mouse apoptotic myoblast cells expose PS and promote cell fusion between healthy myoblasts, which requires mechanical force [93]. The contribution of phagocytosis to dynamic morphogenesis has been shown during *Drosophila* dorsal closure, which requires multiple forces for its completion [94]. These studies suggest that apoptosis-induced phagocytosis may be involved in generating mechanical force in cellular and tissue movement. Such crosstalk or intertwined effects of different cellular behaviors may contribute to non-autonomous apoptosis. Because increases in the tissue tension are caused by overcrowding or the proliferation of neighbors, proliferating cells may be a source of mechanical force in cramped spaces [85,86,87]. Therefore, dissecting the intersections of these cellular interactions and behaviors represents a major direction for future research. Moreover, despite recent progress in understanding cellular communications between apoptotic cells and their neighbors, much remains to be learned about their dialogue in terms of cellular dynamics and molecular conversations. How cells interpret and integrate a diverse range of information from extracellular microenvironments, make appropriate life-and-death decisions and respond to those decisions remain poorly understood. For example, how is apoptosis spatiotemporally regulated in morphogenetic events? How much force is necessary for cells to initiate apoptotic programs? What circumstances decide whether or not apoptotic cells trigger cell proliferation in surrounding cells? Looking ahead, future studies should investigate the precise mechanisms of how apoptotic cells sense specific tissue conditions and select certain interactions with living cells. It will also be important to clarify the relationship between the local and systemic effects of apoptosis. A better understanding of apoptotic communications could provide mechanistic insights into the basic biology during development and homeostasis and lead to potential therapeutic treatments for diseases associated with cellular communication defects.
